# Editorial: Cardiac Microvascular Endothelium Contribution to Cardiac Myocyte Growth, Structure, and Contractile Function

**DOI:** 10.3389/fcvm.2019.00130

**Published:** 2019-09-03

**Authors:** Raffaele Altara, George W. Booz

**Affiliations:** ^1^Department of Pathology, School of Medicine, University of Mississippi Medical Center, Jackson, MS, United States; ^2^Institute for Experimental Medical Research, Oslo University Hospital and University of Oslo, Oslo, Norway; ^3^KG Jebsen Centre for Cardiac Research, University of Oslo, and Center for Heart Failure Research, Oslo University Hospital, Oslo, Norway; ^4^Department of Pharmacology and Toxicology, School of Medicine, University of Mississippi Medical Center, Jackson, MS, United States

**Keywords:** cardiac remodeling, endothelial cell, cardiomyocyte, crosstalk, cell signaling, paracrine action

We have the pleasure of bringing together for the readers of *Frontiers in Cardiovascular Medicine*, three groups of investigators to discuss their interests in the topic of endothelial cell—cardiomyocyte crosstalk in cardiac remodeling. Notably, the three manuscripts offer differing perspectives on the topic, attesting to its complex and dynamic nature. What is clear from the series is the rich interaction between cardiomyocytes and endothelial cells of the heart, reflecting the close physical proximity between the two cell types that is important for normal heart function and health.

Gogiraju et al. provide a comprehensive accounting of the important signaling molecules of both endothelial cells and cardiomyocytes that regulate angiogenesis. These include various growth factors, such as vascular endothelial growth factor (VEGF), placental growth factor (PGF), platelet-derived growth factors (PDGFs), basic fibroblast growth factor (bFGF), epidermal growth factors (EGFs), and hepatocyte growth factor (HGF). This contribution also provides an overview of endothelial transcription factors that regulate angiogenic growth factor expression, as well as a discussion of possible mechanisms for the reduction in angiogenesis with pathological cardiac hypertrophy. These include an imbalance of pro- and anti-angiogenic growth factors, endothelial phosphatases as mediators of angiogenic resistance, endothelial cell death, and epigenetic control of angiogenic gene transcription.

Zeng and Chen focus their contribution on the NAD-dependent deacetylase, sirtuin-3 (SIRT3), which is normally found in mitochondria. These investigators recently reported the novel observation that SIRT3-deficient endothelial cells exhibit decreased basal glycolysis and glycolytic capacity (1), which is associated with increased mitochondrial ROS formation. Notably, in endothelial cells glycolysis and not oxidative phosphorylation is preferentially used to generate ATP to maintain normal cellular functions, including angiogenesis. The review by Zeng and Chen discusses evidence to support the provocative suggestion that impairment of SIRT3-mediated endothelial cell metabolism and angiogenesis may promote cardiomyocyte hypoxia and myocardial fibrosis, leading to diastolic dysfunction and heart failure with preserved ejection fraction (HFpEF). Unresolved is the issue of whether endothelial SIRT3 deficiency or its compromised activity occurs in HFpEF patients, and whether this progresses over time to systolic dysfunction, which is also associated with microvascular rarefaction (2). Also, it would be interesting to investigate whether there is a link between endothelial SIRT3 deficiency and the increased activity of inducible nitric oxide synthase (iNOS) that was recently implicated in the pathogenesis of HFpEF (3).

Zouein et al. provide a brief overview of the importance that the transcription factor STAT3 has been shown to play in the dialog between endothelial cells and cardiomyocytes. This occurs under both normal and diseased states, such as peripartum cardiomyopathy, and involves both genomic and non-genomic actions of this versatile transcription factor. Part of the role of STAT3 in endothelial cell—cardiomyocyte crosstalk involves miRNAs, not only within each cell type, but within endosomal vesicles that are taken up by cardiomyocytes and affect their gene expression profile. These authors also present the novel idea that the non-genomic actions of STAT3 in endothelial cells and cardiomyocytes may serve as a sensor in the heart that fine tunes its response to stress. This sentinel role would involve its translocation to mitochondria, endoplasmic reticulum, and nucleus, as well as its presence at intercalated disc (in the case of cardiomyocytes). The importance of the non-genomic actions of STAT3 *in vivo*, particularly with regard to direct actions in mitochondria, is still controversial (4); although the role of STAT3 in directly regulating mitochondrial function in cardiac myocytes has been questioned on stoichiometric grounds (5), such a role in endothelial cells may be less problematic, since these cells have a lower number of mitochondria. Not specifically addressed in this article is the possibility that nuclear accumulation of unphosphorylated STAT3 (U-STAT3), due to the release of IL-6 from endothelial cells, may act to sustain pressure overload-induced cardiac hypertrophy (6, 7).

Although endothelial cell—cardiomyocyte crosstalk has been extensively investigated, there is more that needs to be explored and understood. Lacking, is an integrated scheme for the crosstalk under physiological and pathological conditions that is based on a systems biology approach. That approach needs to incorporate aspects of the immune system. It needs as well to take into consideration the contribution of metabolism, oxidative stress, neuronal signaling, and other factors in coordinating the dialog. Many questions remain to be answered, but the experimental methods have lagged behind and newer technologies are sorely needed. Hopefully, the ensuing decade will see the development of new experimental approaches and a renaissance in studies investigating this important topic.

## Author Contributions

All authors listed have made a substantial, direct and intellectual contribution to the work, and approved it for publication.

### Conflict of Interest Statement

The authors declare that the research was conducted in the absence of any commercial or financial relationships that could be construed as a potential conflict of interest.
